# Clip-on adapter for simultaneous inline Raman and fluorescence spectroscopy inside semitransparent plastic pipes

**DOI:** 10.1007/s00216-025-06213-3

**Published:** 2025-11-18

**Authors:** L. Hirschberger, K. Wieland, M. Völkl, K. Karaghiosoff, C. Haisch

**Affiliations:** 1 Chair of Analytical Chemistry and Water Chemistry, School of Natural Sciences, Technical University of Munich, Lichtenbergstrasse 4, 85748 Garching, Germany; 2 Competence Center CHASE GmbH, Vienna, Austria; 3 Department of Chemistry, Ludwig Maximilian University of Munich, Munich, Germany

**Keywords:** Raman spectroscopy, Fluorescence spectroscopy, Process analysis, Flow reactor, Inline montoring, Process analytical technology

## Abstract

We present an adapter developed to clip a standard Raman probe (*d* = 9.5 mm) on semitransparent tubes (*d*_OD_ = 0.25″ = 6.35 mm), thus allowing inline spectroscopic measurements in continuous microflow reactor systems. The clip-on adapter can be placed at any position along a fixed or flexible tube, enabling real-time monitoring and control of chemical reactions in the micro-reactor. In addition to Raman scattering, which is detected in reflection mode, the Raman laser (*λ* = 532 nm) also excites fluorescence, detected at a 90° angle relative to excitation. The simultaneous application of Raman and fluorescence spectroscopy is demonstrated by analyzing various vegetable oil mixtures of sunflower and olive oil. The influence of integration time and total flow rate on the signal intensity was investigated on the example of olive oil. Acid-catalytic esterification of ethyl acetate serves as a typical use case for non-contact inline Raman monitoring in a microflow reactor.

**Graphical Abstract:**

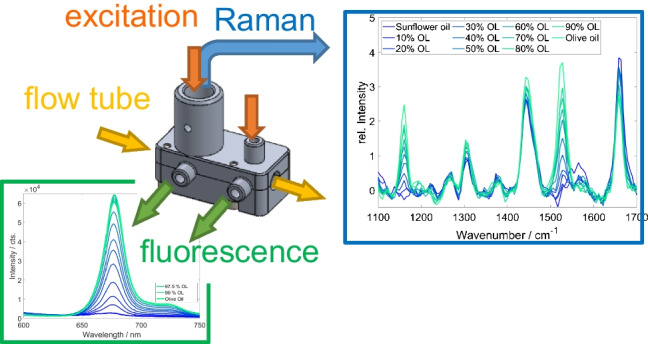

**Supplementary Information:**

The online version contains supplementary material available at 10.1007/s00216-025-06213-3.

## Introduction

Continuous microflow reactors are increasingly used in the chemical and pharmaceutical industry, offering several advantages over conventional batch production, such as improved mixing efficiency, more effective temperature control (especially for exothermic reactions), and increased user safety. The modular design of the reactors allows flexibility in reactor design (e.g., longer mixing channel) and facilitates process scale-up achieved by parallelization, reducing the technical requirements and scale-up effects typically encountered for batch reactors [1–6].

Direct, time-resolved, and non-destructive process and product quality monitoring through process analytical technology (PAT) is crucial for continuous process optimization and control. The chemical industry employs process analytical chemistry (PAC) to characterize a chemical system in an evolving environment, including process development and typical analytical systems in commercial production. In 2004, the US Food and Drug Administration (FDA) introduced PAT as an extension of PAC to improve and control production processes in the pharmaceutical, food, and biotechnology industries while aiming for quality by design (QbD) [7, 8]. PAT allows the optimization of reaction parameters or timely intervention in the process if, e.g., unwanted by-products are formed/detected. Among the spectroscopic PAC tools employed in combination with appropriate data analysis for PAT are (mid and near) infrared (IR), ultraviolet (UV), nuclear magnetic resonance (NMR), fluorescence, and Raman spectroscopy, providing qualitative and quantitative chemical information [3, 9–17].

An in situ spectroscopic system usually comprises either an immersed detection probe (inline) or a flow cell integrated into the product flow. The measuring cell may also be installed as a bypass for online measurements [7]. Inline and online acquisition of analytical data using spectroscopic techniques enables continuous monitoring of the composition of the reaction mixture, including hazardous or sensitive intermediates, at different positions in the reactor setup and aggressive chemical media. The combined use and analysis of different (orthogonal) spectroscopic or other physical sensors, such as pressure, temperature, or pH sensors, allow a wide range of chemical and physical properties to be measured and may also be useful to deepen the understanding of the process at hand [3, 7, 13, 14, 18].

The in situ application of fiber-coupled spectroscopic PAT becomes particularly useful for hazardous chemical reaction monitoring as it eliminates the need for sampling including the risk of potential sampling artifacts [19]. While inline configurations with direct contact between the probe and analyte place high material requirements on the probe, resulting in higher costs, this is less critical in non-contact versions.

Raman spectroscopy is an optical spectroscopic technique often employed for qualitative and quantitative applications in process control and reaction monitoring. Non-invasive, label-free analysis detecting specific molecular fingerprint information of organic and inorganic materials and real-time capability due to short data acquisition times typically in the range of seconds to minutes belong to the most promising aspects of the technique. While water shows strong absorption in the mid-IR spectral region, the Raman scattering effect of hydroxyl bonds is weak, making it particularly useful for the analysis of aqueous reaction mixtures [11, 14, 15].

Different approaches have been proposed for the integration of Raman spectroscopy into microflow applications. One critical parameter to achieve the optimum signal-to-noise ratio is the geometrical adjustment between the probe optics and the sample flow. Nelson et al*.*demonstrated that Raman spectroscopy can be employed to analyze varying concentrations of aqueous nitric acid solutions in commercial microfluidic cells with two different Raman probes [20]. Chaplain et al. used a self-designed PTFE flow cell with a quartz window to apply Raman spectroscopy [21]. The probe was positioned anterior to a 90° channel bend with the focal point in the flow channel. It was used to monitor the heterogeneously catalyzed Suzuki cross-coupling reaction between 4-bromobenzonitrile and phenylboronic acid. Ferstl et al*.*reported on a cylindrical flow cell with a reflective coating on the opposite side of the probe. The reflection of the beam increased the overall detected light intensity [3]. Pelletier et al*.*used a Raman probe within the product output stream in combination with partial least squares (PLS) as a multivariate calibration model to quantitatively follow the ozonolysis of trans-stilbene in a continuous ozonolysis reactor. Also, the impact of process parameters such as reactant flow rate and ozonator voltage on the product quality was evaluated by inline Raman spectroscopy [19].

The presence of fluorophores hampers the application of Raman spectroscopy as it evokes a strong background signal, which may even impede any Raman detection due to detector saturation. However, the fluorescence signal can also be used as a source of chemical information, e.g., for identifying biochemical components such as chlorophyll in vegetable oils. Fluorescence spectroscopy is a standard technique in chemical analysis, featuring extreme sensitivity with a detection limit down to the nanomolar or picomolar range. Fluorescence signals are usually several orders of magnitude stronger than Raman signals. Raman spectroscopy, on the other hand, is more specific. While Raman spectroscopy is commonly performed in a 180-degree geometry between excitation and detection, the fluorescence signal is typically detected using a perpendicular arrangement [9, 10, 14, 16, 22]. Fluorescence spectroscopy is primarily implemented in microflow reactors to control the synthesis of fluorescent nanomaterials. Monitoring non-fluorescent analytes requires an additional labeling step with dyes such as rhodamine or fluorescein in the reactor [14, 23]. The laser wavelength in the visible range used for Raman spectroscopy can also be employed for the excitation of fluorescence. The implementation of simultaneous fluorescence and Raman spectroscopy increases the flexibility of the integrated metrology, possibly providing a second line of evidence for reliable inline monitoring.

The present work reports on a custom-made probe adapter for a microflow reactor system, enabling simultaneous inline fluorescence and Raman spectroscopy as highlighted by a selection of applications shown here. The direct attachment and flexible positioning of the adapter to a tube of the flow system aim to provide a low-cost option for inline and real-time measurements.

## Methods

### Materials

Ethanol (> 99.8%, Carl Roth GmbH, Germany), acetic acid (> 99.8%, Carl Roth GmbH, Germany), ethyl acetate (> 99.8%, Carl Roth GmbH, Germany), and sulfuric acid (96%, Merck KGaA, Germany) were used as supplied. The extra virgin olive oil, produced in Italy, and the sunflower oil (made in Hungary) were purchased in local stores in Germany. The samples were stored in the dark at room temperature and were used without further purification.

### Instruments

For Raman spectroscopy, a commercial setup was used equipped with a 532-nm CW-laser (gem 532, output power, 100 mW, Laser Quantum, Santa Clara, USA), a spectrometer (QE-Pro 532 nm, FFT-CCD detector, slit, 50 μm, spectral resolution, 0.66 nm OceanInsight, Orlando, USA), and a Raman probe (RPB532ff, InPhotonics, Norwood, USA). The fiber-coupled SPEC STD UV/Vis/NIR spectrometer (sarspec, Vila Nova de Gaia, Portugal), covering the spectral range from 185 to 1100 nm, was used for fluorescence spectroscopy. The Raman setup CW-laser (532 nm, output power, 50 mW) was used as the fluorescence excitation source for the analysis of vegetable oils using the Raman probe.

### Measurement setup

#### Vegetable oil analysis

Two programmable syringe pumps Standard Infuse/Withdraw Pump 11 Pico Plus Elite (Harvard Apparatus, Cambridge, USA), 10-mL plastic syringes Omnifix® LuerLockSolo (Braun, Melsungen, Germany), Swagelok PFA 1/4″ tubings, and a plastic Tee-mixer (PEEK) were employed for the flow experiments with the vegetable oils. The adapter was installed on the PFA tubing at 30 cm from the Tee-mixer for the measurement. Figure S1 in the Supporting Information shows a schematic of the flow reactor setup.

#### Acid-catalyzed esterification

Two Cetoni MESYS Mid Pressure syringe pump modules (Korbußen, Germany) with 25-mL glass syringes were operated in a timed continuous mode for the acid-catalyzed esterification synthesis for each reagent feed. The reactor consists of three Corning Advanced Flow Low Flow reactor plates (2× SJH, 1× RTH, Corning, NY, USA). The plates were temperature-controlled using a Julabo Dyneo DD-BC12 thermostat (*T*_1_, Seelbach, Germany) and a Julabo FCW2500T chiller (*T*_2_, Seelbach, Germany). Ethanol (EtOH) and conc. acetic acid (AcOH) containing sulfuric acid (1% v/v) as a catalyst were used as the feed solutions. The optical adapter was installed on the PFA tubing near the reactor outlet, resulting in a total reactor volume of 3.58 mL (see Figure S2). The reactor volume is defined as the volume between the first contact of the reactant streams on the first plate and the probe adapter after the third reactor plate.

### Data processing

Data analysis was performed in MATLAB (R2023b, The MathWorks Inc., Natick, USA). The Raman spectra were stored as ASCII files in the spectral range between 70 and 4700 cm^−1^ by OceanView (OceanInsight, Orlando, USA). The spectral range between 300 and 4000 cm^−1^ was selected for data processing. The Raman spectra for the test applications were baseline-corrected using the asymmetrically reweighted Penalized Least Squares algorithm (parameter: *λ* = 10^5^ and *p*= 0.01) [24]. Fluorescence spectra were detected between 530 and 750 nm and used without further processing.

## Results and discussion

### Clip-on adapter design and characterization

**Fig. 1:**
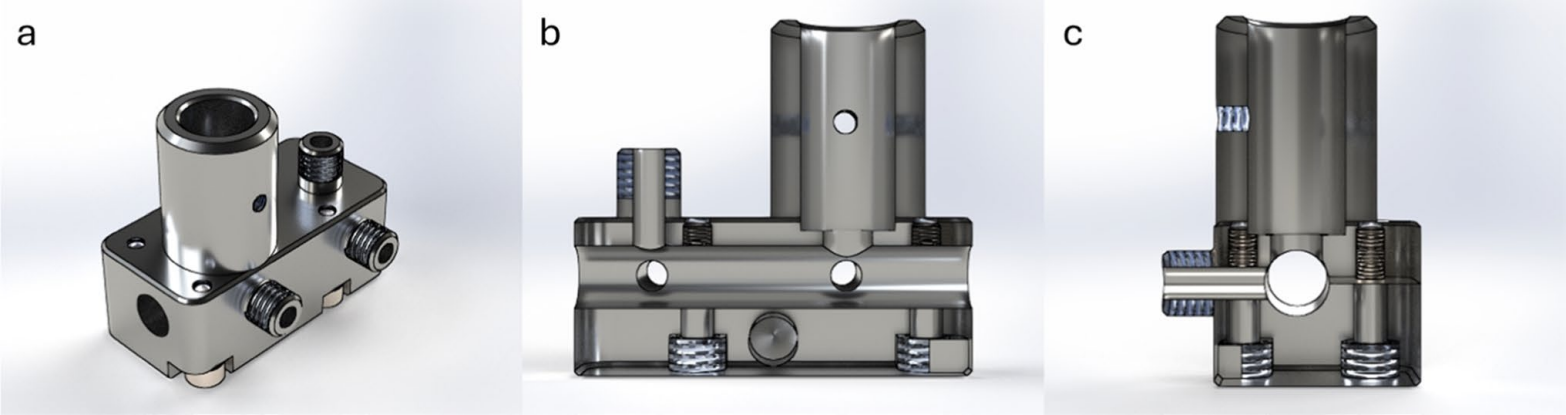
The rendering of the clip-on adapter, which consists of a front plate with a Raman probe holder, a fiber connector, and a back plate: **a** rendering of the assembled adapter, **b** longitudinal cross-section, and **c** transverse cross-section

A flow cell inserted into the process stream is the most common approach for integrating a spectroscopic inline process monitoring technique. This additional component in the setup must be considered in the reactor design (effect on flow profile, total volume, etc.). Here, we chose a less invasive alternative solution by measuring straight through the semitransparent PFA tube. While facing an inevitable reduction in signal intensity, this arrangement enables us to measure at any desired point along the flow system and allows flexible re-positioning without stopping the process or opening the liquid system. The technical realization is straightforward: The two sides of the holder are put around the tube and closed by four M3 screws. A commercial non-contact Raman probe is then fixed such that its optical axis is directed radially into the tube (see Fig. 1). The installation is completed within minutes. The probe adapter allows flexible analysis and monitoring in highly corrosive or hazardous environments as the probe does not come into contact with the reaction mixture; hence, circumventing the need for cost-intense, chemically resistant materials such as Hastelloy steel as a probe material [25].

An optically transparent material with minimal scattering and Raman activity is the preferred tube material for non-contact measurements to achieve maximum signal intensity. Quartz is usually the material of choice. However, glass tubing cannot be used for more chemically demanding reaction settings such as high pressure or hazardous chemicals. For these applications, perfluoroalkoxy alkanes (PFA) are used as alternative tubing and fitting materials in flow chemistry because of their high-temperature resistance, mechanical flexibility, and resistance to solvents and aggressive chemicals such as strong acids [26]. In this work, the effect of PFA as a less transparent material on the Raman signal of the reaction mixture in the tube was investigated [25].

**Fig. 2:**
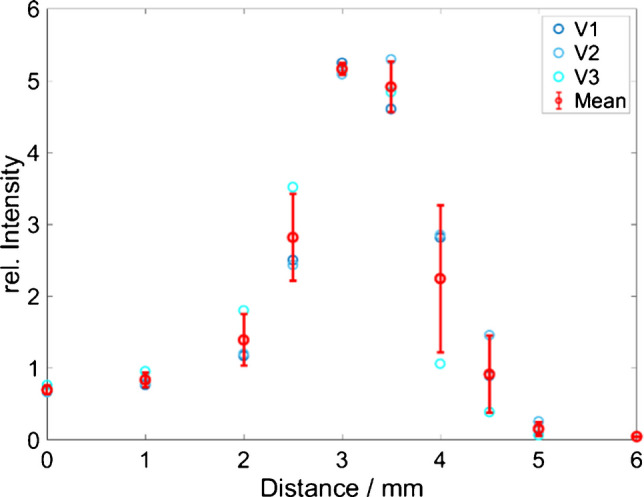
Intensity ratio *I*_EtOH_/*I*_PFA_ depending on the working distance (error bar, 1 σ with *n* = 3)

According to the manufacturer’s specifications, the focal length of the used probe lens is 7.5 mm in air. The laser focal length changes according to Snell’s law when measuring through the tube materials due to their differing refractive indices (*n*_quartz_ = 1.46, *n*_PFA_= 1.35) [27, 28]. Additionally, the refractive index of the liquid inside the tube impacts the focusing [25]. The optimal working distance *d*_w_ leading to the maximum signal collected from the liquid inside the PFA tube was determined using ethanol as a sample. The *d*_w_ between the probe and the tube wall was varied between 0 and 6 mm in steps of 1 mm; between 2 and 5 mm, the step size was reduced to 0.5 mm. The intensity of the ethanol band at 888 cm^−1^ (C-C-O stretching) and the intensity of the PFA band at 736 cm^−1^(C-C stretching) were evaluated to determine the ratio of both signals at various distances [25, 29, 30]. The optimum position, featured by the highest analyte signal and the lowest tube signal, was found to be at *d*_w_ 3.0–3.5 mm. Figure 2 shows the ratio of the signal intensities *I*_EtOH_/*I*_PFA_ depending on the working distance.

The optimum working distance for the Raman probe, fixed in the adapter, was *d*_w_ = 3 mm. The adapter was tested on quartz glass tubes as well as PFA tubes with a continuous flow of *F* = 1 mL/min of pure ethanol. In general, the signal intensities of the Raman spectra measured in the glass tube were more intense than those measured in the PFA tube. The respective Raman spectra contain bands of both components, the tube material PFA (736 cm^−1^ and 1384 cm^−1^) and ethanol (888 cm^−1^) (see Figure S3). The quartz bands were not visible. However, the background signal in the spectra collected in the quartz capillary is higher than that measured in the PFA tube. After baseline correction, the maximum band intensity of ethanol at 888 cm^−1^ measured in the PFA tube is 67 ± 1.1% of the same band in the quartz tube, indicating a reduction of the analyte band intensity by 1/3 in the less transparent PFA tube (see Figure S3). Similar results were observed by J. Wang et al. [25]

**Fig. 3:**
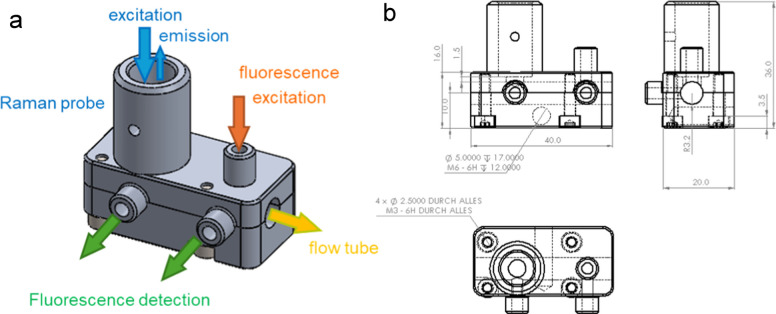
**a** Principle schemes and **b** technical drawing of the designed adapter

The fiber optical Raman probe combines Raman excitation and detection on one optical axis. For strongly fluorescent samples, detecting a Raman signal is hardly possible due to the saturation of the highly sensitive Raman detector. Hence, a fluorescence detection channel was integrated into the clip-on fiber-probe holder. The Raman excitation laser (*λ* = 532 nm) excites the fluorescence, while detection is performed via an additional optical fiber (SMA 905) installed orthogonally to the excitation light. No further collection optics for the fluorescence are installed (see Fig. 3).

A separate measuring position for fluorescence spectroscopy is integrated into the adapter platform. It is offset by 10 mm to the combined Raman/fluorescence probe position along the tube. The excitation light is coupled into the tube via fiber optics; detection is again carried out orthogonally via fiber optics with connections for SMA 905. Both fibers are used without additional optics (see Fig. 1). The initial test setups were produced by PVA by fused filament fabrication (FFF) 3D printing. The final version was produced using anodized aluminum. It makes mechanical strong and precise production comparably easy. Anodization leads to a chemical and mechanical surface resistance. Of course, for even more demanding applications, production by stainless steel or PEEK (polyetheretherketone) is also possible. The mechanical precision of our aluminum version is so that repeated uninstalling and re-installation do not reflect on the reproducibility of the Raman measurement. That means that repeated installation always induces signal fluctuations below the fluctuations of the measurements itself.

### Optimization of measurement parameters

The optical integration time *T*_int _needs to be optimized for each application individually to fully exploit the dynamic range of the optical detector while avoiding saturation [31]. Also, the mathematical averaging needs to be balanced between the maximum signal-to-noise ratio and the measurement frequency required to follow the dynamics of concentration variations. In continuous flow reactor systems, a steady state is observed at a specific position along the reactor tubes once the system is optimized and equilibrated. Deviations from this steady state must be detected quickly, limiting the maximum integration time to seconds or minutes.

#### Characterization of measurement parameters in a continuous flow

Olive oil as a highly fluorescent sample was chosen as a model analyte for the simultaneous inline Raman and fluorescence spectroscopy. Cooking oils, such as olive and sunflower oil, are certainly not typical samples in microflow reactor systems; however, continuous monitoring of these two components, for instance as quality control during bottling, is critical as the product quality of expensive olive oils and adulteration with low-price sunflower oils are common concerns in the food industry [33].

Fluorescence spectroscopy is suitable for sensitively detecting specific additives or contaminants (e.g., chlorophyll in sunflower oil). However, quenching effects have been considered at high fluorophore concentrations, resulting in a reduced signal intensity. Raman spectroscopy, however, has proven to be a valuable tool for evaluating specific chemical properties of olive oil, such as total unsaturation, lipid oxidation, and carotenoid content. Raman spectra can provide comprehensive information about the fatty acid composition of the blend based on the chemical-specific spectral fingerprints [32, 33].

The Raman laser (*λ* = 532 nm) was used as a common excitation source for fluorescence and Raman spectroscopy, while we employed two separate spectrometers for fluorescence and Raman detection. The optimum integration times have been determined separately for both spectroscopic techniques. The laser power was set to 50 mW to avoid the saturation of the Raman detector due to the high fluorescent signal components in vegetable oils.

**Fig. 4:**
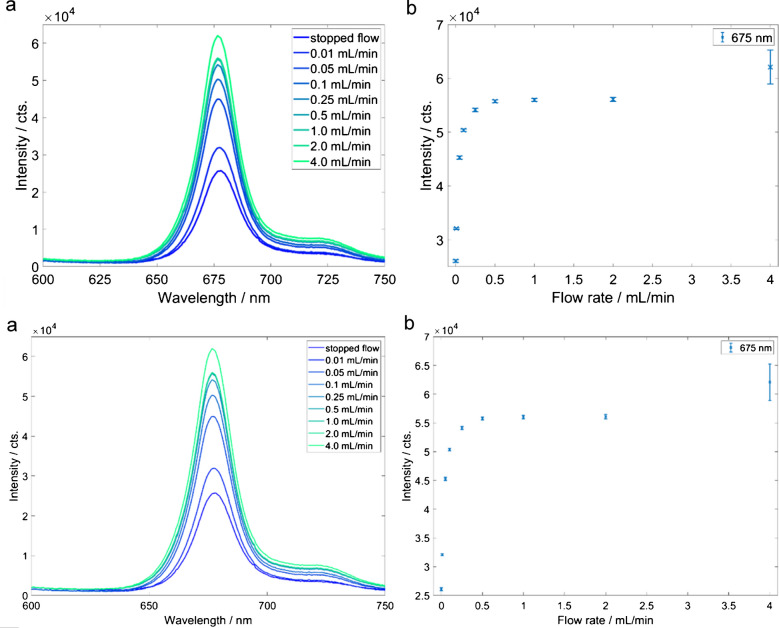
**a** Fluorescence spectra of olive oil in PFA tubing at different total flow rates, *λ*_ex_ 532 nm, *T*_int_: 200·150 ms. **b** Intensity maximum of the fluorescence peak at 675 nm as a function of the total flow rate (error bar, 1 σ, *n* = 200)

The maximum intensity of the broad signal peak at 675 nm in the fluorescence spectra of olive oil was analyzed. According to the literature, the signal can be assigned to the pigments of the chlorophyll group. This group includes chlorophylls a and b and the phaeophytins a and b [34]. An integration time *T*_int_ = 150 ms was chosen for further measurements (see Figure S4). At higher integration times, saturation of the detector was reached. The olive oil was measured in stopped-flow mode and at different flow rates to investigate the influence of the flow rate on the fluorescence intensity (see Fig. 4). As we did not expect high temporal variations, we decided to average 200 spectra, each with an integration time of 150 ms, resulting in a 30-s averaging time. While the integration time has to be optimized to fit the dynamic range of the detection system, mathematical averaging of the individual spectra reduces the amount of data and the noise. The number of averages is chosen to fit the desired temporal resolution.

The lowest fluorescence signal was measured in stopped-flow mode. Continuous measurement of the same sample volume showed a fast loss of signal intensity. We attribute this observation to photobleaching. Even a very low flow rate of 0.01 mL/min already leads to increased fluorescence. At higher flow rates of up to 4 mL/min, the signal from the olive oil further increases. The higher flow rate leads to a faster exchange of the fluorophore in the focal volume. At a flow rate of 0.1 mL/min, a molecule in the flow takes approximately 1.41 s to pass the focal volume at the most narrow waist (189 µm). At 0.25 mL/min, 0.5 mL/min, 1 mL/min, and 2 mL/min, the transit time drops to 563 ms, 282 ms, 141 ms, and 70 ms, respectively (see Fig. 4).

The intensity of the Raman spectra increases with increasing integration time, measured at a constant flow rate *F* = 1 mL/min, showing a strong background, which leads to detector saturation at an integration time of *t*_Raman_ = 1.5 s (see Figure S5). The integration time *t*_Raman_ = 1 s was chosen for further measurements, accounting for a possible background increase due to fluorescence effects in the various oil mixtures.

**Fig. 5:**
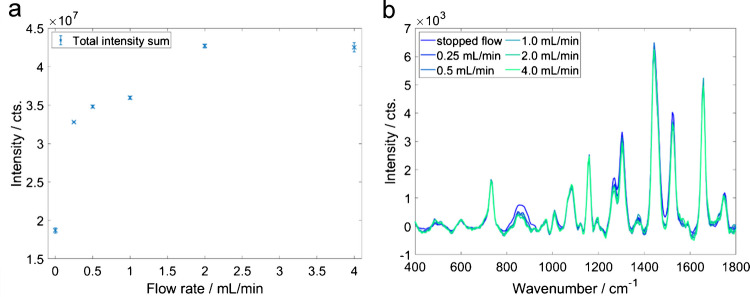
**a** Area under the curve of the Raman spectrum prior to baseline correction as a function of total flow rates (error bar 1 σ, *n* = 30). **b** Baseline-corrected Raman spectra of olive oil for different flow rates

As in fluorescence spectroscopy, the background in the Raman spectra of the olive oil increases with increasing flow rate (see Figure S6). This background can be attributed to fluorescence affecting the Raman measurement. Hence, a robust background subtraction procedure is required. All the Raman spectra of the olive oil, measured at various flow rates, were baseline-corrected to allow quantitative evaluation of the Raman bands (see Fig. 5b). The background-corrected spectra feature only minute differences.

### Application of inline fluorescence and Raman spectroscopy for olive oil and sunflower oil mixtures

The monitoring and discrimination of various vegetable oil blends in a continuous flow (*F* = 1 mL/min) served as a test application for parallel inline fluorescence and Raman spectroscopy. Pre-mixed solutions were used to ensure the homogeneity of the oils in the flow channel. The mixing ratio was changed in steps of 10 v/v %. Raman and fluorescence spectra were collected for each mixture using the integration times determined beforehand (see previous section). Again, fluorescence and Raman spectra were averaged over 30 s (200 · 150 ms resp. 30 · 1 s). Five technical replicates of the measurements were generated for a statistical evaluation.

**Fig. 6:**
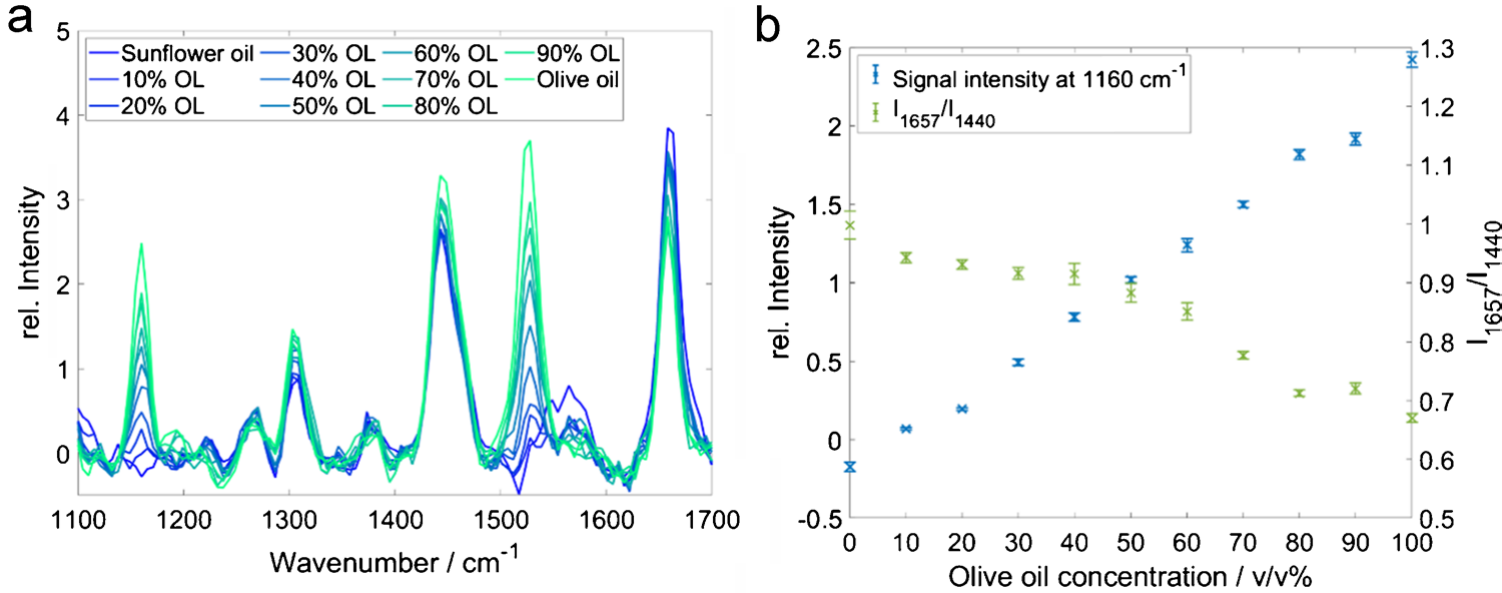
**a** Raman spectra of various sunflower/olive oil mixtures (OL, olive oil); *F* = 1 mL/min. **b** Raman band intensities at 1160 cm^−1^ and 1527 cm^−1^ of sunflower/olive oil mixtures and the band ratio *I*_1657_/*I*_1440_ (unsaturated/saturated fatty acids) as a function of olive oil content in the oil mixture (flow rate *F* = 1 mL/min; error bars 1 σ, *n* = 5)

The Raman bands at 1160 cm^−1^ and 1527 cm^−1^ exhibit the most pronounced change as a function of concentration and, thus, were further investigated as marker bands to distinguish between the blends (see Fig. 6a). Both bands can be assigned to beta-carotene, an antioxidant component of high-quality olive oils (1160 cm^−1^: C-C stretching; 1527 cm^−1^: C=C stretching) [35]. Despite the low concentration of the pigment in the oil, carotenes can be detected with the excitation wavelength of 532 nm because of the signal enhancement caused by the Raman resonance effect [36–38]. As carotene is only contained in the olive oil but not in the sunflower oil, we can use these ß-carotene bands for quantification of olive oil content. The band at 1160 cm^−1^ features a linear correlation with the olive oil content. Below a concentration of 20 v/v%, the carotene bands are not visible in the spectrum.

The band at 1657 cm^−1^ (C=C stretching, polyunsaturated) is related to unsaturated fatty acids, and the one at 1440 cm^−1^(C‒H bending) indicates saturated fatty acids in the oils [35, 39]. The *I*_1657_/*I*_1440_ ratio between the unsaturated and the saturated fatty acids reveals the fatty acid compositions (see Fig. 6b). Olive oil has a higher content of saturated fatty acids and a lower content of unsaturated fatty acids. The highest band ratio *I*_1657_/*I*_1440_ is obtained with pure sunflower oil and the lowest with olive oil. The ratio between the two bands decreases with higher proportions of olive oil, as the content of polyunsaturated fatty acids in olive oil is lower than in sunflower oil. The combined analysis of *I*_1657_/*I*_1440_and the carotene bands supports the detection of adulterations, i.e., the addition of sunflower oil or olive oils of inferior quality to high-quality olive oil [35, 40].

The fluorescence intensity was evaluated based on the signal intensity at 675 nm. This peak stems primarily from chlorophyll and is present only in pure olive oil (see Figure S7) [34, 41].

**Fig. 7:**
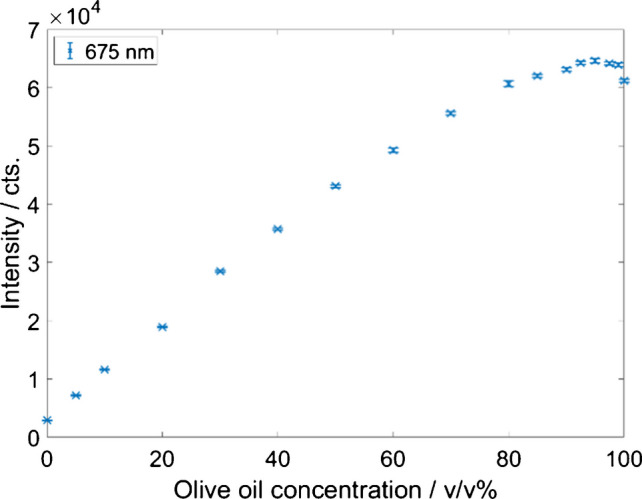
Fluorescence signal intensity maximum between 650 and 700 nm from various sunflower/olive oil mixtures as a function of the olive oil content (error bar, 1 σ, *n* = 5)

The fluorescence peak maxima of the different oil blends are summarized in Fig. 7, while the corresponding fluorescence spectra covering the spectral range from 650 to 700 nm are depicted in Figure S7, with an olive oil–specific maximum of 675 nm. For increasing olive oil concentrations of up to 90 v/v%, the signal intensity increases. Up to a content of 50 v/v% olive oil, a linear correlation between the intensity of the signal at 675 nm and the olive oil concentration in the mixture can be observed. However, at concentrations starting from 50 v/v%, the slope decreases. Above 90 v/v% olive oil, the signal intensity reaches a plateau (see Fig. 7). High concentrations of chlorophyll in olive oil can lead to self-absorption and quenching of the fluorescence by the formation of less/no fluorescent dimers [34, 42]. This use case highlights the multi-sensor advantage leveraging the benefit of one technique while the other one reaches its limits and vice versa. The combination of fluorescence and Raman spectroscopy allows covering the complete dynamic range of the olive/sunflower oil mixtures.

Additional information can be extracted using a second fluorescence excitation wavelength in the range of 400 nm. Sunflower oil can be characterized by a fluorescence signal in the range of 430 to 520 nm caused by the higher linoleic acid content [41]. The adapter platform is designed to allow an additional light source to be integrated at an additional optical access. With the excitation source used here (532 nm), Raman spectroscopy is better suited than fluorescence spectroscopy to detect impurities in olive oil by analyzing the carotene bands and the ratio of saturated to unsaturated fatty acids in continuous flow. Fluorescence spectroscopy is more suitable for sensitively detecting low concentrations of fluorescent contaminants, such as added chlorophyll, in sunflower oil.

### Reaction monitoring of the acid-catalyzed esterification in microflow reactor

After establishing the fundamental parameters, we ventured to a typical application for online process monitoring, i.e., the optimization of process conditions in a continuous flow system. Our model system for this test was the acid-catalytic esterification of acetic acid (AcOH) and ethanol (EtOH) to ethyl acetate (EtOAc) in a microflow reactor setup (volume, 3.58 mL). For this task, we employed only Raman spectroscopy. The volume concentration of the sulfuric acid catalyzing the reaction was 1 v/v%. Two separate thermostats were used to control the reactor temperature (*T*_1_ and *T*_2_). The first reactor plate was temperature-controlled separately from the other two plates (see Figure S2). To quantitatively determine the product concentrations from the chemical reaction, various mixtures of the reactants and the product were measured in the absence of a catalyst. The Raman spectrum (*t*_Raman_ 1 s) was normalized to the PFA signal maximum at 736 cm^−1^. The individual pure substance spectra were used as a reference to assign the bands in the mixtures (see Figure S8). The bands at 789 cm^−1^ and 847 cm^−1^ were assigned to the CH_2_ rocking and C–C symmetric stretch vibrations of ethyl acetate.^43^ The band at 888 cm^−1^ was identified as the C–C symmetric stretch vibration of ethanol, and the bands at 680 cm^−1^ and 893 cm^−1^ are evoked by the O=C–O bending and C–C symmetric stretch vibrations of acetic acid.^44^ For calibration, the signal intensity of ethyl acetate at 789 cm^−1^ was determined at different concentrations. The product concentration in the solution and the resulting yield were determined using the linear correlation between the product concentration and the ethyl acetate band at 789 cm^−1^ (see Figure S9). The molar reactant ratio EtOH/AcOH was 1:2 for all experiments. Five Raman spectra (60·times 1 s) of the reaction stream under different reaction conditions were used to determine the product’s concentration and yield under various conditions.

**Fig. 8:**
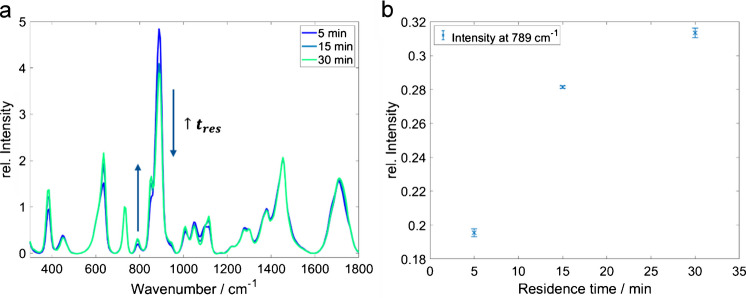
**a** Raman spectra of the reaction solution as a function of the residence time. Reaction conditions: molar reactant ratio (EtOH/AcOH) 1:2; *T*_2_ 80 °C; error bar 1 σ, *n* = 5. **b** Ethyl acetate intensity (band at 789 cm^−1^) as a function of the residence time

At a constant temperature *T*_2_ = 80 °C, the residence time in the reactor was varied by changing the flow rate. Reduced residence times result in lower product concentrations (see Fig. 8 and Table 1). However, depending on the intended application, either the relative efficiency, given as product concentration, has to be optimized or optimization aims for a maximum absolute production efficiency, i.e., product mass per time. Then, the trade-off between lower flow rate, i.e., throughput, and higher efficiency has to be determined. Here, the key advantage of the online monitoring system becomes evident: this optimization can be performed as fast as the reactor adjusts to the stepwise set conditions; the resulting efficiency is revealed in real-time.

**Table 1:** EtOAc concentration and yield (including standard deviation, SD) depending on the residence time determined using the calibration (see Figure S9). Reaction condition: molar reactant ratio (EtOH/AcOH), 1:2, *T*_2_ 80 °C

Residence time (min)	EtOAc (mol/L)	SD (mol/L)	Yield in %	SD in %
5	2.61	0.014	46	0.2
15	3.57	0.004	62	0.1
30	3.88	0.017	68	0.3

**Fig. 9:**
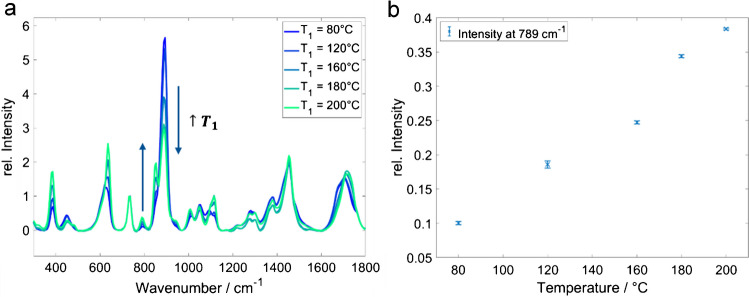
**a** Raman spectra of the reaction mixture after passing the reactor at different temperatures *T*1_1_. **b** Band intensity at 789 cm−1 as a function of the reactor temperature *T*_1_. Reaction conditions: molar reactant ratio (EtOH/AcOH) 1:2; *T*_2_ 80 °C; residence time 5 min, error bar 1 σ, *n* = 5

The temperature *T*_1_ of the first reactor plate was varied between 80 and 200 °C. Higher temperatures resulted in higher product concentrations, as seen in Table 2 and Fig. 9. The combination of reactor temperatures *T*_1_ = 200 °C and *T*_2_ = 80 °C resulted in the highest product signal intensities and yield. The residence time was set to 5 min for this study in order to reduce the consumption of chemicals.

**Table 2:** Ethyl acetate concentration and yield (including standard deviation, SD) at different reactor temperatures *T*_1_. Reaction conditions: molar reactant ratio (EtOH/AcOH) 1:2; *T*_2_ 80 °C; residence time 5 min

Temperature (°C)	EtOAc (mol/L)	SD (mol/L)	Yield in %	SD in %
80	1.59	0.016	28	0.5
120	2.52	0.056	44	1.0
160	3.18	0.024	55	0.4
180	4.23	0.024	74	0.4
200	4.66	0.012	81	0.2

## Conclusion

We present a clip-on adapter for the flexible integration of Raman and fluorescence spectroscopy in microfluidic reactors. The adapter allows direct, non-contact measurements on a PFA tube in a microflow reactor system. The influence of the critical measurement parameters, integration time, and flow rate was identified for the inline measurement to obtain an optimum signal intensity. Before transferring to a new application, these parameters must be adapted to the respective conditions, such as possible fluorophores. In addition, baseline correction has been shown to allow the comparison of spectra with background signals of varying intensity without affecting the analyte signal after processing. Various sunflower and olive oil mixtures have been characterized in parallel measurements with the two spectroscopic techniques using the same excitation source.

The results highlight the benefits of the multi-sensor concept demonstrating that combining both spectroscopic techniques provides valuable chemical-specific information by leveraging the strengths and limitations of each technique, while the experimental effort is limited. While the proposed clip-on sensor leads to limited sensitivity due to the non-optimum optical access, it has significant technical advantages for real-world applications. It can be installed essentially anywhere along a process stream; it can even be shifted quickly between several positions. As the investment for the clip alone is much lower than that of the spectroscopic instrumentation, it is also reasonable to install several clips and move only the Raman and fluorescence fiber probes between clips. The limited sensitivity of the Raman detection renders our clip-on approach, with the detection through the tube, less suitable for trace detection. However, in process optimization, especially in microflow reactor designs, the aim is often a precise analysis of the main components, i.e., educts and products present at higher concentrations, a task perfectly suiting our combined online spectroscopy approach.

## Supplementary Information

Below is the link to the electronic supplementary material.

Supplementary Material 1 (PDF 556 KB)

## Data Availability

All relevant data is presented in the manuscript.
