# Supplementary material for: Clip-on adapter for simultaneous inline Raman and fluorescence spectroscopy inside semitransparent plastic pipes
Source: Anal Bioanal Chem. 2025 Nov 18;418(1):167–77. doi: 10.1007/s00216-025-06213-3 (PMC12774953; doi:10.1007/s00216-025-06213-3)
Supplement: Supplementary file 1 — Supplementary Material 1 (PDF 556 KB) [file 216_2025_6213_MOESM1_ESM.pdf]

Supporting Information

Clip-on adapter for simultaneous inline Raman and fluorescence spectroscopy inside semitransparent plastic pipes

L. Hirschberger[a]; K. Wieland[b]; M. Völkl[c], K. Karaghiosoff[c]; C. Haisch[a*]

a) Chair of Analytical Chemistry and Water Chemistry, School of Natural Sciences, Technical University of Munich, Garching, Germany.

b) Competence Center CHASE GmbH, Vienna, Austria.

c) Department of Chemistry, Ludwig Maximilian University of Munich, Munich, Germany

Corresponding author
Christoph Haisch

Chair of Analytical Chemistry and Water Chemistry,

School of Natural Sciences

Technical University of Munich

Lichtenbergstrasse 4

D-85748 Garching, Germany

haisch@tum.de

Instrumental

Schematics of the continuous flow setups

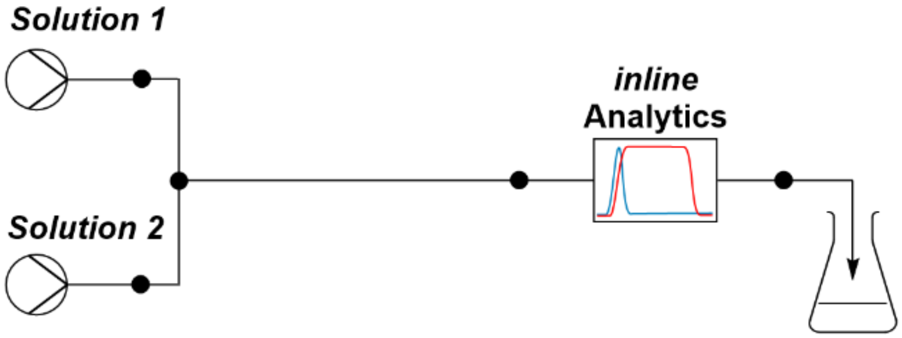

Figure S1: General setup scheme of a simple flow reactor system with two pumps and a PFA tube (Ø: ¼ inch) for testing inline Raman and fluorescence spectroscopy.

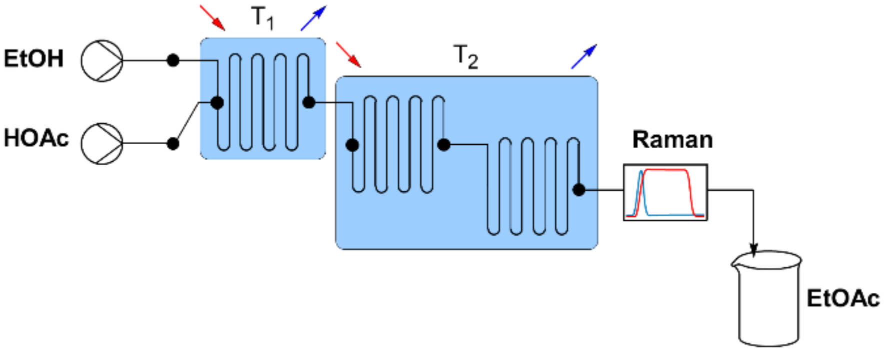

Figure S2: General setup scheme of a flow reactor system for the acid-catalyzed esterification to ethyl acetate, consisting of two syringe pumps, three reactor plates, and two thermostats, connected by a PFA tube (Ø: ¼ inch, 6.35 mm).

Experimental

Comparison of the Raman spectra of ethanol in a quartz and a PFA tube

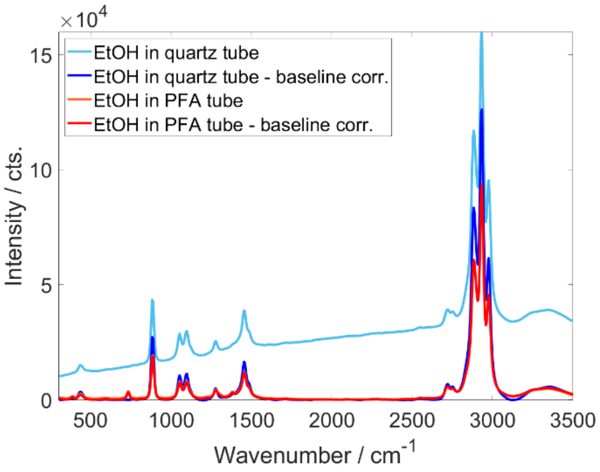

Figure S3: Raw and baseline-corrected Raman spectra of ethanol in a quartz glass and PFA tubing. (T_{int}: 1 s).

Determination of measurement parameters for olive oil

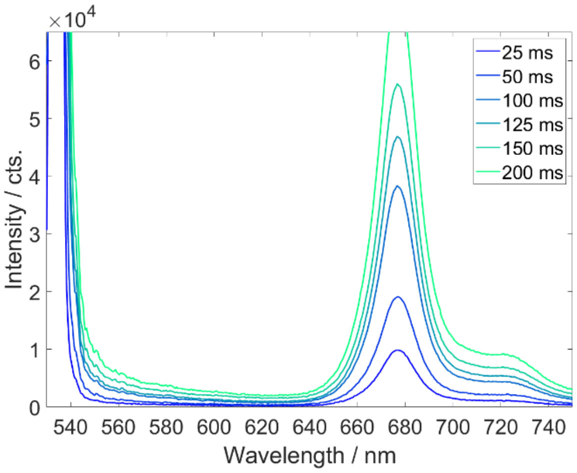

Figure S4: Fluorescence spectra of olive oil in PFA tubing for different integration times with a constant total flow rate (F = 1 mL/min), excitation wavelength: 532 nm.

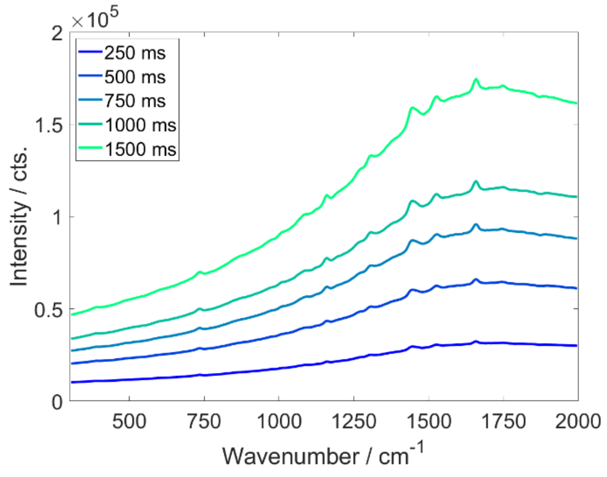

Figure S5: Raw Raman spectra of olive oil at a constant flow rate (F = 1 mL/min) for different integration times.

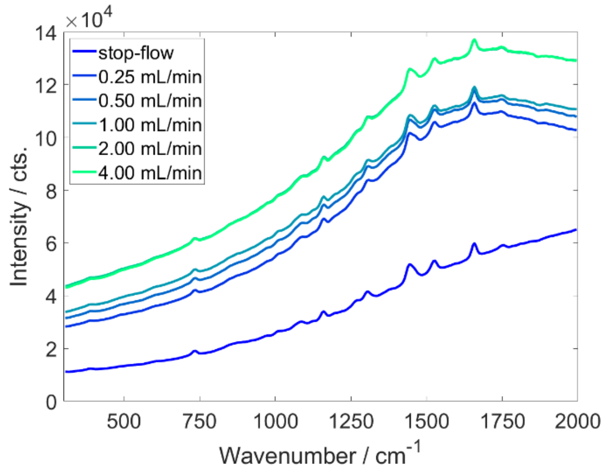

Figure S6: Raw Raman spectra of olive oil for different flow rates (T_{int} = 1 s).

Spectroscopic measurements of oil blends

Fluorescence spectroscopy

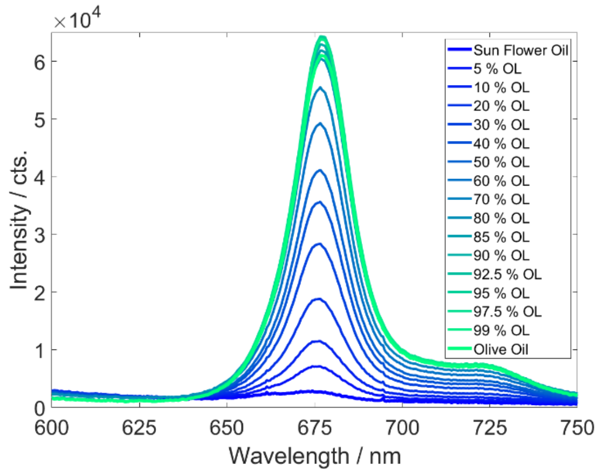

Figure S7: Fluorescence spectra of sunflower and olive oil (OL) blends. Excitation wavelength: 532 nm. Integration time: 150 ms; total flow rate: 1 mL/min.

Acid catalytic esterification of ethanol and acetic acid to ethyl acetate

Reference Raman Spectra

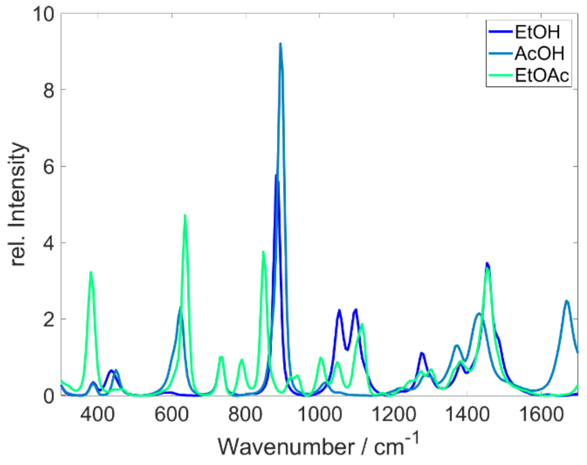

Figure S8: Raman Spectra of ethanol, Acetic Acid, and Ethyl acetate measured in a PFA tube under continuous flow rate.

Different concentrations of ethyl acetate in the tube stream

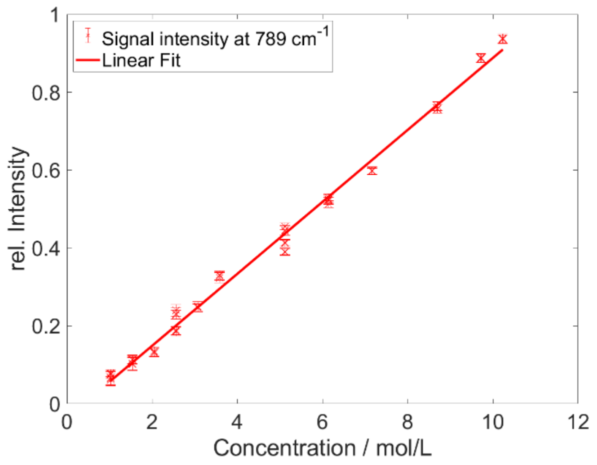

Figure S9: Intensity maximum at 789 cm^{-1} normalized to PFA band as a function of ethyl acetate concentration and the resulting linear fit curve (y = 0.0922·x - 0.0464).
